# Parameter optimization of double‐blade normal milk processing and mixing performance based on RSM and BP‐GA

**DOI:** 10.1002/fsn3.1198

**Published:** 2019-09-13

**Authors:** Jiangtao Qi, Wenwen Zhao, Za Kan, Hewei Meng, Yaping Li

**Affiliations:** ^1^ College of Mechanical and Electrical Engineering Shihezi University Xinjiang China; ^2^ Laboratory of Northwest Agricultural Machinery Ministry of Agriculture Xinjiang China

**Keywords:** BP‐GA algorithm, mixing, normal milk processing, parameter optimization, pasteurization

## Abstract

Temperature stability was taken as the evaluation index of processing performance, and the three factors that influence normal milk processing and mixing performance were optimized by response surface analysis and BP‐GA neural network algorithm. Analysis results showed the influence order of the factors on temperature stability was as follows: shape > height > rotating speed. In the optimization by response surface methodology (RSM), when rotating speed was 30 r/min, height was 31 mm, and blade shape was a full trapezoid, predicted value and actual value of variable coefficient were 0.0046 and 0.0044 respectively, with relative error of 4.5%. In the optimization by BP‐GA neural network algorithm, when rotating speed was 34 r/min, height was 25 mm, and blade shape was a full trapezoid, the predicted value and actual value of variable coefficient were 0.0036 and 0.0035 respectively, with relative error of 2.9%. The predicted root‐mean‐square error of the model by the BP‐GA neural network algorithm was 0.0013, determination coefficient was 0.9960, and relative percent deviation was 8.4961, which showed better performance than the RSM model. Thus, the BP‐GA neural network algorithm has better fitting performance, and then, the optimal working parameter combination was confirmed, which could provide reference to improving double‐blade normal milk processing and mixing device design and milk processing quality.

## 1 INTRODUCTION

Dairy industry is an important part of modern agriculture. It is of great significance to adjust the structure of agricultural industry, develop rural economy, increase farmers' income, and improve the quality of the whole people. At present, cattle farm management is developing rapidly in the direction of scale, standardization, and intensification, and calves are the reserve force of cattle farms. Calf raising serves as an important basis of ensuring sustained productivity and production benefit of pasture land (Zhou, Zhang, Liu, He, & Liang, 2018). Precise feeding of calves is one of the steps in calf raising, and normal milk is an essential approach for calves to obtain nutrition. The quality of normal milk is essential to boosting growing ability of calves and reducing morbidity (Shi, 2018; Wu, 2016). Efficient and high‐quality normal milk processing could ensure precise feeding of calves, and temperature change during processing is an important factor influencing normal milk processing quality. Therefore, determination of working parameters is of great significance to ensure the working performance of the normal milk processing and mixing device.

Response surface methodology (RSM) and Artificial Neural Network (ANN) as nonlinear optimization methods are frequently applied in structural optimization design of machine components and optimization of working parameter combination. For example, Winiczenko, Górnicki, Kaleta, and Janaszek‐Mańkowska (2018) and Winiczenko, Górnicki, Kaleta, Martynenko, et al. (2018) optimized the ANN topology for predicting the rehydrated apple cubes color change using RSM and GA. The result turned out that the optimal ANN topology can be considered as more precise for predicting color change in the rehydrated apple cubes. Samuel and Okwu (2019) simulated and optimized the productive rate conditional parameter of coconut oil ethyl esters and did test verification by using RSM and ANN. Pilkington, Preston, and Gomes (2014) extracted artemisinin from artemisia annua using RSM and ANN and optimized combination of extraction temperature, extraction time, and solvent parameters. Sinha, Chowdhury, Saha, and Datta (2013) established a prediction model for simulation and optimization of dye extraction by RSM and ANN. Muthusamy, Manickam, Murugesan, Muthukumaran, and Pugazhendhi (2019) extracted pectin from helianthus annuus (sunflower) heads using RSM and ANN modeling by a genetic algorithm approach. Yu, Huang, Lin, Kuo, and Shieh (2019) used Artificial Neural Networks and Response Surface Methodology to compare the effection of ultrasound‐assisted extraction of chlorogenic acid from lonicera japonica. The above research showed that RSM and ANN can establish the extraction process model very well, and the optimization effect of the ANN is better in the process of optimizing the extraction condition parameters. In addition, some scholars used the response surface method and the artificial neural network to finish the research on the machining performance of martensitic stainless steel, surface roughness of keyway milling, surface finish analysis of wire electric discharge machined specimens and cutting parameters in turning of gray cast iron. On the basis of modeling, the machining performance of mechanical materials was analyzed and optimized, and the experimental verification was carried out (Bhupinder & Misra, 2019; Ghosh, Mandal, & Mondal, 2019; Laouissi, Yallese, Belbah, Belhad, & Haddad, 2019; Zerti, Yallese, Zerti, Nouioua, & Khettabi, 2019).

Tang, Huang, and Fang (2011) did optimal design on face plate structure of the numerical control rotary table by applying genetic algorithm and BP neural network. Yang, Zhan, Wu, Wang, and Bao (2015) did optimal design on the cold extrusion dies of bearing inner race on automobile hubs based on orthogonal test, BP neural network, and genetic algorithm. Du, Li, Wang, Wu, and Lin (2017) did size optimization on chief bars of small‐wing flank‐type forest fruit collection device using genetic algorithm. Chen, Xue, et al. (2015) optimized the shape of boom truss and the cross‐sectional dimensions of spray boom by multi‐island genetic algorithm. Chen, Li, Cao, and Zheng (2015) established a multi‐objective optimization mathematical model taking vertical vibration acceleration and elevation angular acceleration as indexes by applying genetic algorithm. Ma, Li, He, Meng, and Liang (2006) studied the performance of stripping elements on sugarcane harvester based on BP neural network. Dong, Zhu, et al. (2017) studied the performance parameters of black tea fermentation using extreme learning machine algorithm. Yang et al. (2018) optimized the root‐lifting speed of cassava harvester based on improving spider cluster algorithm. Wang, Dong, Wu, and Fang (2017) optimized corn planting density and fertilization quantity based on BP neural network. Gao et al. (2017) optimized the vacuum freeze‐drying process of bitter melon slices based on genetic algorithm. The above research showed that the neural network algorithm could complete the optimization of the mechanism parameters and the working performance parameters combination.

Therefore, based on the research methods mentioned above, taking the designed double‐blade ordinary milk processing and mixing device as test platform, and temperature stability as evaluation index for processing performance, the parameters of the ordinary processing and mixing device, such as rotating speed of agitator blades, height from agitator blades from barrel bottom, shape of the blades, were optimized by using RSM and BP‐GA. At last the optimal parameter combination was obtained and the optimization method for the device was determined, through comparison analysis, which offered reference to improving design of normal milk processing and mixing device and quality of processed milk.

## 2 MATERIALS AND METHODS

### 2.1 Materials and equipment

#### 2.1.1 Overall structure of the normal milk processing and mixing device and working principle

**Figure 1:**
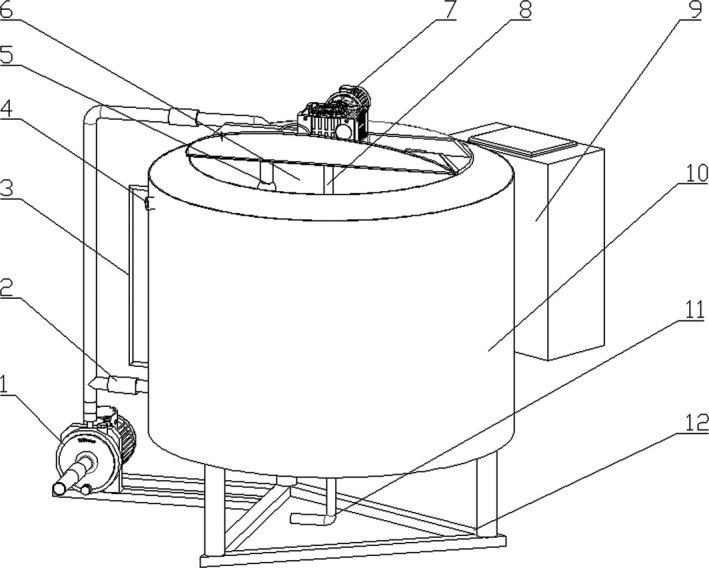
Schematic diagram of the structure of mixing device for normal milk processing. The main components are as follows: 1. Water pump, 2. Solenoid valve, 3. Liquid level meter, 4. Water outlet, 5. Cleaning nozzle, 6. Interior barrel for processing and storage, 7. Mixing electric motor, 8. Mixing shaft, 9. Electric cabinet, 10. Exterior barrel for water circulation, 11. Milk supply pipeline, and 12. Body frame

The overall structure of the normal milk processing and mixing device is shown in Figure 1. The device is mainly composed of an interior barrel for processing and storage, an exterior barrel for water circulation, a mixing electric motor, a mixer and a water pump. The device can accomplish milk pasteurization through heating and cooling by water circulation.

In working process, the mixing device for constant milk processing mainly mixes normal milk through a motor‐driven mixer and improves temperature conductivity, including heating and cooling of normal milk. Water bath is adopted in heating: first, raw milk is poured into the interior barrel, and then, the water pump pumps water to the exterior barrel, in which the heating rod heats the water. Temperature is transferred to milk through barrel wall, when the detected milk temperature by temperature sensor reaches the set value, the heating process is stopped. The cooling process adopts the method of water circulation, after pumping water to the exterior barrel, water temperature gradually reduces and cools the milk through barrel wall, and then, the discharged hot water flows into water storage tank through water outlet for reutilization; when milk temperature reduces to the set value, the normal milk processing is finished.

#### 2.1.2 Temperature detection system

**Figure 2:**
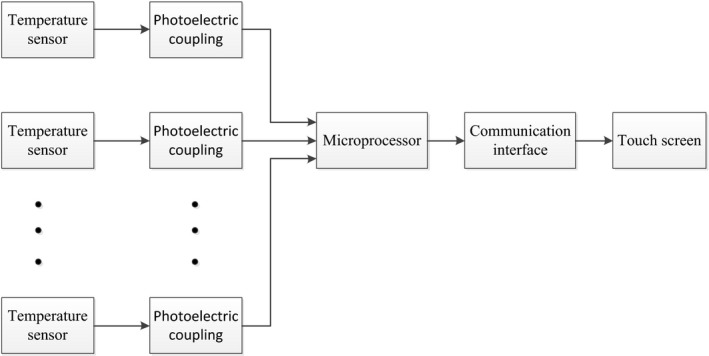
Block diagram of milk processing testing platform. It is composed of temperature detection module, single‐chip microcomputer minimum system and human–computer interaction module

Normal milk processing testing platform is composed of temperature detection module, single‐chip microcomputer minimum system and human–computer interaction module, as is shown in Figure 2. It mainly measures temperature at different positions during normal milk processing, with sampling frequency of 1 Hz and 12 collection points, temperature measurement range of −55 to 125°C. Collected data are stored by a USB flash disk. A touch screen is used in operation system.

The system microprocessor is an 8‐bit STC89C54RD+microprocessor produced by STC micro Technology Co., Ltd. By considering precision of temperature measuring in processing and collection of temperature data at different heights, there were three groups of temperature sensors, each group having four sensors. The length of the first group sensors was 436 mm, that of the second group sensors was 287 mm, and that of the third group sensors was 138 mm.

Temperature sensors were DS18B20 monobus temperature sensors. In order to improve the anti‐interference ability of the system, an opto‐isolator unit was added in the testing platform by using Omron optocoupler relay G3VM‐402C; and the TK6071iQ type touch screen produced by WEINVIEW CO., LTD was adopted to display and store temperature data. RS‐485 communication network was used to connect the touch screen and single‐chip microcomputer. Configuration software was used to make “project files,” which can be downloaded to the touch screen through PC and communication port of the touch screen; the programming software of the touch screen was EB8000 and the collected data can be transferred to Excel and stored to the USB flasher disk.

**Figure 3:**
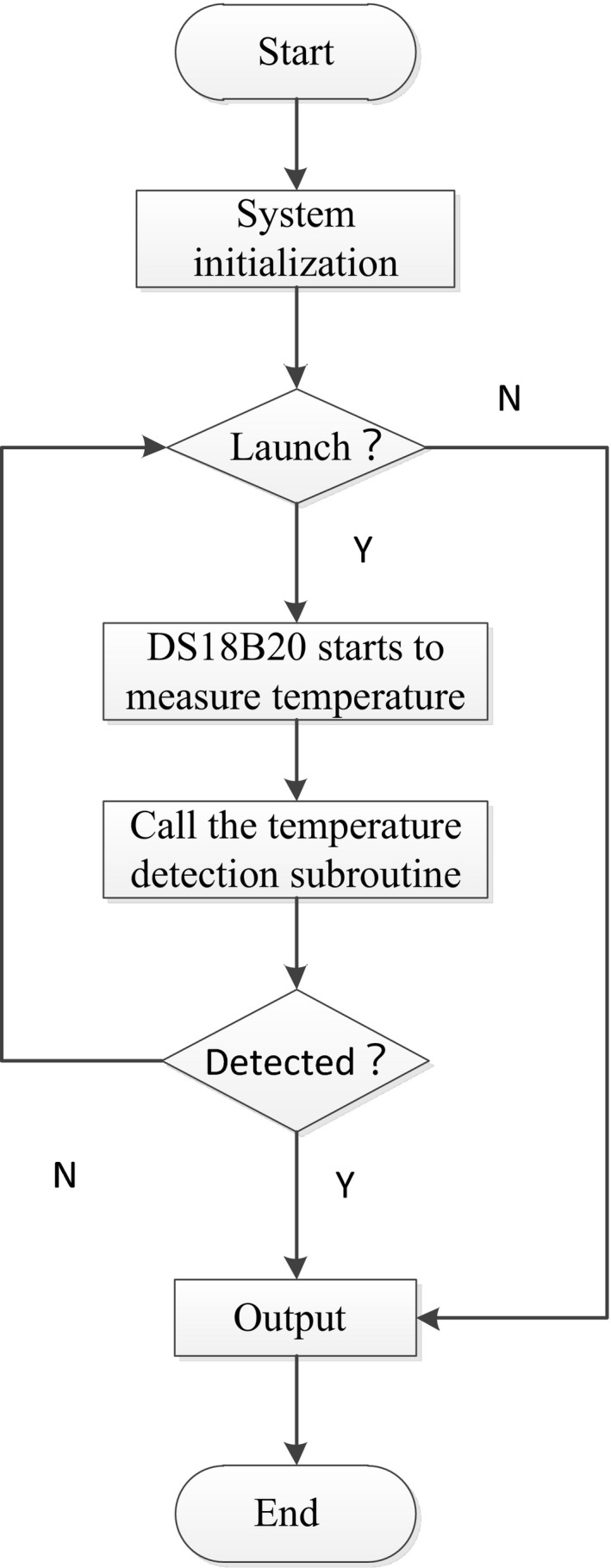
Flowchart of the main program of the processing testing platform

**Figure 4:**
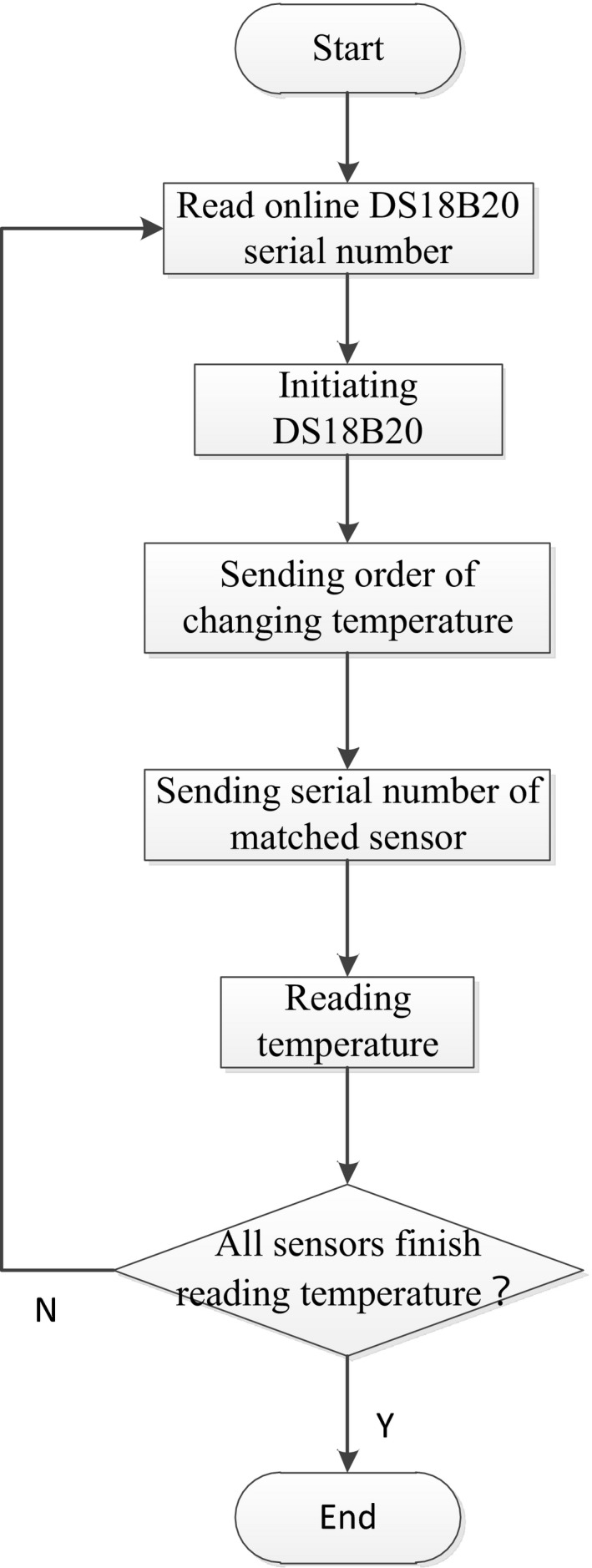
Flowchart of the temperature detection program of the processing testing platform

The software system is a distributed structure, in which the single‐chip microcomputer mainly realizes reading of real‐time temperature data, the touch screen stores and displays data, and they are connected through serial port. The flowchart of master control program of the single‐chip microcomputer and the temperature detection flowchart are shown in Figures 3 and 4, respectively.

#### 2.1.3 Sample collection and equipment

The milk used in the test was produced in the third cattle farm of 121 regiment, Eighth Division of Xinjiang Production and Construction Corps, the mixing device for constant milk processing (self‐designed), temperature detection system (self‐designed), laptop (win10), agitator blades of three shapes (full trapezoid, full rectangular, and trapezoid frame, which were self‐designed).

### 2.2 Test design and method

#### 2.2.1 Test design

A three‐factor and three‐level test was designed by taking rotating speed of agitator blades, height from agitator blades to barrel bottom, shape of agitator blades as level factors, and temperature stability as the evaluation index. The code table of test factors are shown in Table 1.

**Table 1:** Factor level coding form

Codes	Factors		
	Rotating speed (r/min)	Height (mm)	Shape
−1	25	20	Full trapezoid
0	35	30	Full rectangular
1	45	40	Trapezoid frame

#### 2.2.2 Evaluation index

Temperature stability in this test is described by variable coefficient. The smaller the variable coefficient is, the less the degree of variation of data under this level combination would be, showing better heating and cooling stability of the material processing system. Variable coefficient is calculated by Equation 1.

(1) CV=S/V

where CV is variable coefficient;

*S* is the standard deviation of samples;

*V* is the mean value of samples.

#### 2.2.3 Establishment of BP‐GA integrated optimization algorithm

**Figure 5:**
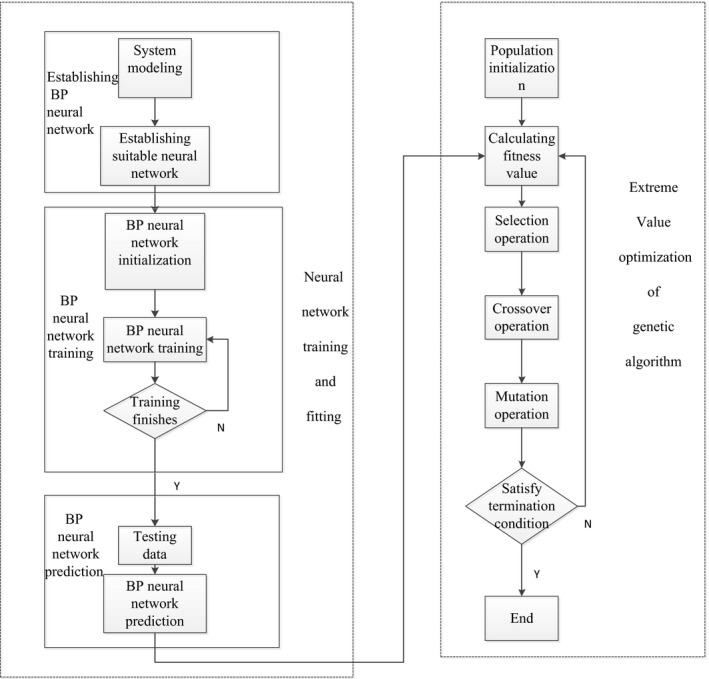
Flowchart of BP‐GA algorithm. BP‐GA integrated optimization algorithm mainly has two steps, training and simulation of BP neural network and extreme value optimization of genetic algorithm

Back Propagation (BP) neural network algorithm is a kind of neural network of feedforward learning algorithm and back propagation algorithm, which can effectively solve the problem of connection weight of hidden layer in the multi‐layer neural networks and improve the self‐learning and organization ability of neural network (Liu, Wu, Wang, Tan, & Wang, 2012; Zhang et al., 2014). Genetic Algorithms (GA) is a random and parallel search optimization method that simulates the natural genetic mechanism and biological evolution theory. This method introduces the biological evolution theory of “survival of the fittest” and has the advantages of high efficiency, parallel, and global search (Duan, Zhang, Wei, Xiao, & Wang, 2017; Wang, Sun, et al., 2017). BP‐GA integrated optimization algorithm mainly has two steps, training and simulation of BP neural network and extreme value optimization of genetic algorithm. Training and fitting of neural network mainly includes establishing BP neural network, which is trained by inputting and outputting data with a nonlinear function, and prediction of function output. The extreme value optimization process of genetic algorithm is mainly the prediction result of neural network as the individual fitness value, and the global optimal value and the corresponding input value are found through selection operation, cross operation and mutation operation. The flowchart of the algorithm is shown in Figure 5.

The specific steps of the BP‐GA integration optimization algorithm are as follows:

##### Population initialization

The individual coding method is a real code, and each individual is a real string, which is composed of input layer and hidden layer connection weight, hidden layer threshold, hidden layer and output layer connection weight, and an output layer threshold. The individual weight and the threshold value of the individual are initialized.

##### Fitness function

According to the initial weight and threshold of BP neural network obtained by individuals, the output of BP neural network is trained with training data, and the absolute value of error between prediction output and expected output is taken as individual fitness value *F*. The formula is as follows:

(2) $$F=k\left(\sum_{i=1}^{n}\text{abs}(y_{i}-o_{i})\right)$$

where *n* is output points for the network; *y*_i_ is the expected output of the *i* node. *o*_i_ is the predicted output of the *i* node, *k* is the coefficient.

##### Selection operation

The genetic algorithm operation selection roulette method, which is a selection strategy based on fitness ratio. The selection probability *p_i_* of each individual *i* is as follows:

(3) $$f_{i}=k/F_{i}$$

(4) $$p_{i}=\frac{f_{i}}{\sum_{j=1}^{N}f_{i}}$$

where *F*_i_ is the fitness value of individual *i*, *N* is the number of population individuals.

##### Cross operation

The cross operation method adopts real number cross operation, chromosomal *a_k_* of position *k,* and chromosomal *a_l_* of position *l* cross operation at the position *j* is as follows:

(5) $$\left.\begin{array}{c} a_{\mathrm{kj}}=a_{\mathrm{kj}}\left(1-b\right)+a_{\mathrm{li}}b\\ a_{\mathrm{li}}=a_{\mathrm{li}}\left(1-b\right)+a{}_{\mathrm{kj}}b \end{array}\right\}$$

where *b* is a random number between [0, 1].

##### Mutation operation

Variation of *a_ij_* about the *j* gene of individual *i*, the methods of mutation operation are as follows:

(6) $$a_{\mathrm{ij}}=\left\{\begin{array}{cc} a_{\mathrm{ij}}+\left(a_{\mathrm{ij}}-a_{\max}\right)\times f\left(g\right) & r>0.5\\ a_{\mathrm{ij}}+\left(a_{\min}-a_{\mathrm{ij}}\right)\times f\left(g\right) & r\leq 0.5 \end{array}\right.$$

(7) $$f\left(g\right)=r_{2}{\left(1-g/G_{\max}\right)}^{2}$$

where *a*_max_ is the upper bound of the gene, *a*_min_ is the lower bound of the gene, *r*_2_ is a random number, *g* is the number of iterations, *G*_max_ is the maximum number of evolution, and *r* is the random number between [0, 1].

## 3 RESULTS AND ANALYSIS

A three‐factor and three‐level test was designed by taking rotating speed of agitator blades, height between agitator blades and barrel bottom, shape of agitator blades as level factors, and temperature stability as the evaluation index. There were 17 test points, including 12 analytic factors, and 5 zero errors of estimate. Based on the principle of milk pasteurization, in this test, the milk with volume of 90 L and normal temperature as initial temperature was heated to 80°C and cooled to 39°C. In the process, the designed temperature detection system was used to obtain the real‐time temperature. In the process of data processing, the temperature values on the 12 sensors of the temperature detection system were exported, and 20 time points were randomly selected and the data of 12 sensors corresponding to each time point were extracted, namely a data matrix of 20 × 12 was extracted. The extraction process requires that every two time intervals should be equal, and the 20 sets of data include the whole process of temperature rise and fall. The data of 12 sensors corresponding to each time point were processed to calculate the coefficient of variation, and the 12 coefficients of variation were averaged to obtain the coefficient of variation, and the stability of temperature change was evaluated. Test scheme and response values are shown in Table 2.

**Table 2:** Test scheme and response values

Test No.	Test scheme			Variable coefficient *Y*	RSM Predicted value	BP‐GA Predicted value
	Rotating speed(r/min)/*X*_1_	Height (mm)/*X*_2_	Shape (double‐blade)/*X*_3_			
1	−1	−1	0	0.0391	0.0384	0.0390
2	1	−1	0	0.0427	0.0408	0.0425
3	−1	1	0	0.0350	0.0364	0.0344
4	1	1	0	0.0242	0.0244	0.0241
5	−1	0	−1	0.0177	0.0156	0.0174
6	1	0	−1	0.0101	0.0091	0.0143
7	−1	0	1	0.0254	0.0268	0.0253
8	1	0	1	0.0210	0.0236	0.0209
9	0	−1	−1	0.0149	0.0172	0.0146
10	0	1	−1	0.0054	0.0055	0.0067
11	0	−1	1	0.0288	0.0276	0.0286
12	0	1	1	0.0242	0.0208	0.0240
13	0	0	0	0.0085	0.0100	0.0101
14	0	0	0	0.0089	0.0100	0.0102
15	0	0	0	0.0092	0.0100	0.0102
16	0	0	0	0.0114	0.0100	0.0103
17	0	0	0	0.0123	0.0100	0.0103
Predicted root‐mean‐square error					0.0018	0.0013
*R* ^2^					0.9772	0.9960
Relative percent deviation					6.3094	8.4961

### 3.1 Response surface model and significance testing

Multiple regression fitting analysis was done on the test results in Table 2 by applying Design‐Expert 9 (developed by Stat‐Ease) and the quadratic polynomial regression model of the coefficient of variation *Y* to the independent variables (*X*_1_, *X*_2_, *X*_3_) is as follows:

(8) $$\begin{array}{c} Y=0.010-2.413\times 10^{-3}X_{1}-4.588\times 10^{-3}X_{2}+6.425\times 10^{-3}X_{3}-3.6\times 10^{-3}X_{1}X_{2}+8.25\times 10^{-4}X_{1}X_{3}\\ +1.225\times 10^{-3}X_{2}X_{3}+0.013X_{1}^{2}+0.012X_{2}^{2}-4.23\times 10^{-3}X_{3}^{2} \end{array}$$

The significance of the influence of each variable on indexes in the regression equation is judged by *F* test. The smaller the probability *F* is, the higher the significance of the corresponding variable will be (Irna, Jaswir, Othman, & Jimat, 2018; Irungu et al., 2019). The variance analysis results of response surface model in Table 3 show that, the regression model *p *< .0001, indicating that the model was extremely significant; the Lack of fit *p* (.0964) was higher than .05, which was not significant, indicating that within the test range, the predicted values of the regression model fitted the actual values, determination coefficient of the variable coefficient *R* ^2^ was 0.9772, showing that the regression model could explain the 97.7% variability of test data and the predicted values were highly correlated with actual values, and this model can be used to analyze and predict temperature stability.

**Table 3:** Variance analysis results of response surface model

Source	Squares	*df*	Square	*F* value	*p* value	Significance
Model	2.050 × 10^–3^	9	2.278 × 10^–4^	33.31	<.0001	Significant
*X*_1_	4.656 × 10^–5^	1	4.656 × 10^–5^	6.81	.0349	*
*X*_2_	1.684 × 10^–4^	1	1.684 × 10^–4^	24.62	.0016	**
*X*_3_	3.302 × 10^–4^	1	3.302 × 10^–4^	48.30	.0002	**
*X*_1_ *X*_2_	5.184 × 10^–5^	1	5.184 × 10^–5^	7.58	.0284	*
*X*_1_ *X*_3_	2.722 × 10^–6^	1	2.722 × 10^–6^	0.40	.5481	
*X*_2_ *X*_3_	6.002 × 10^–6^	1	6.002 × 10^–6^	0.88	.3800	
$X_{1}^{2}$	6.786 × 10^–4^	1	6.786 × 10^–4^	99.24	<.0001	**
$X_{2}^{2}$	6.574 × 10^–4^	1	6.574 × 10^–4^	96.14	<.0001	**
$X_{3}^{2}$	7.534 × 10^–5^	1	7.534 × 10^–5^	11.02	.0128	*
Residual	4.786 × 10^–5^	7	6.838 × 10^–6^			
Lack of fit	3.653 × 10^–5^	3	1.218 × 10^–5^	4.30	.0964	Not significant
Pure error	1.133 × 10^–5^	4	2.833 × 10^–6^			
Cor total	2.098 × 10^–3^	16				

* *p *< .05 (significant).

** *p *< .01 (highly significant).

### 3.2 Analysis on influence effect of factors

**Figure 6:**
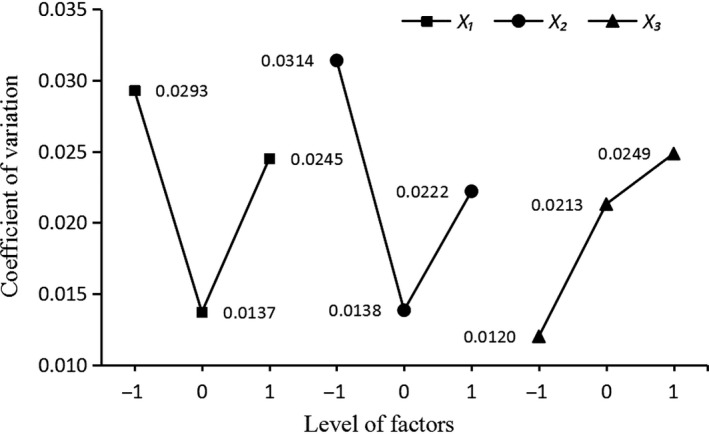
Relationship between factor levels and coefficients of variation. *X*_1_ means rotating speed of agitator blades; *X*_2_ means height from agitator blades to barrel bottom; *X*_3_ means shape of agitator blades

**Figure 7:**
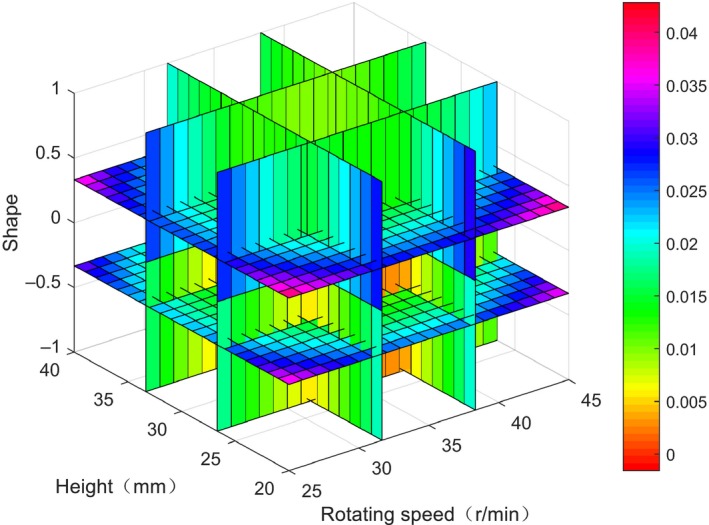
Contourslice between variable coefficient and rotating speed, blade shape and height

In order to directly show the influence of the three factors on variable coefficient, the horizontal index relationship shown in Figure 6 is obtained by taking the horizontal change of the factors as the transverse coordinate and the average value of the coefficient of variation as the longitudinal coordinate. At the same time, a contour slice was drawn by MATLAB R2016a (by Math Works of USA) according to Equation 8. A contour slice was drawn on six coordinate points, 32 and 39 r/min for *x*_1_, 27 mm and 34 mm for *x*_2_, and −0.3 and 0.4 for *x*_3_ (as is shown in Figure 7), and the color of the slice and grids describes the values of variable coefficient. Table 2 and Figure 5 show that the overall influence trend within the set parameters is: as the stirring paddle speed increases, the coefficient of variation first decreases and then increases. When the speed is 30–40 r/min, the coefficient of variation is small, namely the temperature of the milk is stable. The coefficient of variation is better; when the height of the paddle blade is 25–35 mm from the bottom of the barrel, the coefficient of variation is small; when the shape of the paddle blade is trapezoidal full face and rectangular face, the coefficient of variation is small.

**Figure 8:**
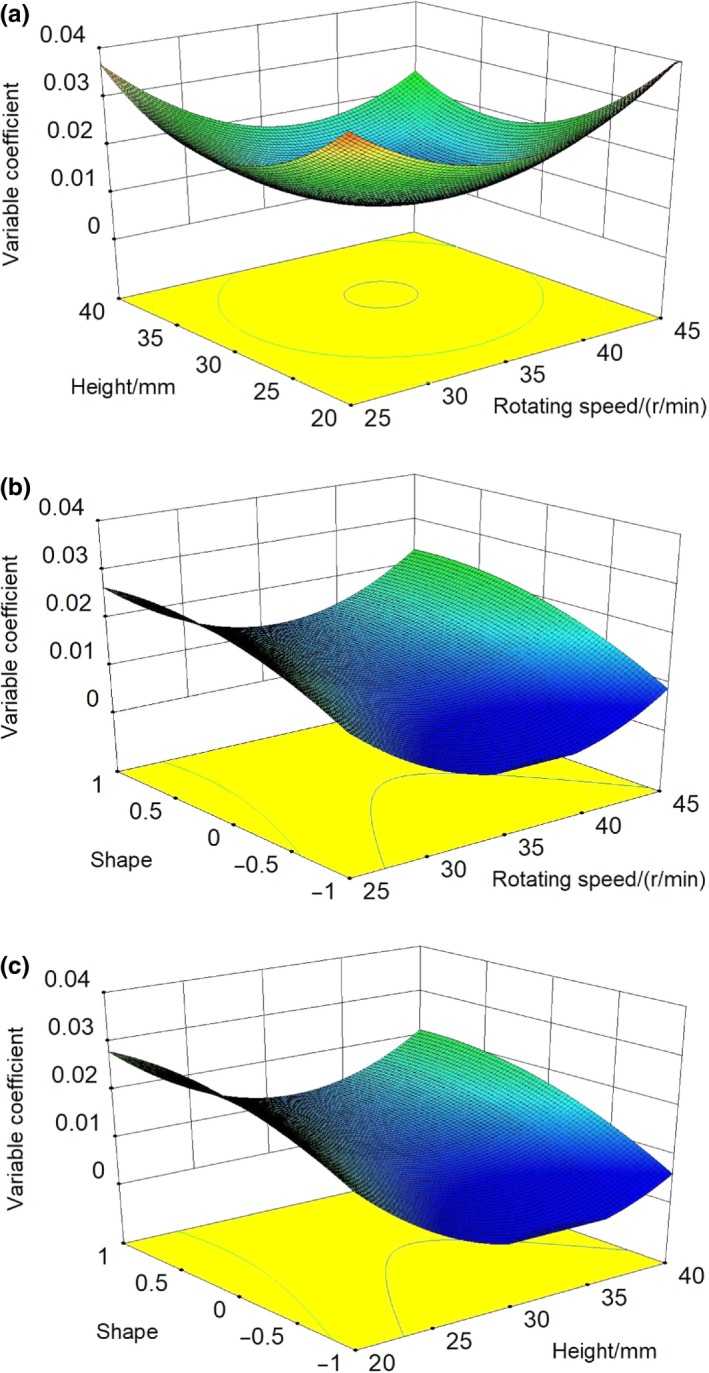
Response surface of interaction effect between the three factors and variable coefficient. (a) shows the interaction between height and rotating speed affects the coefficient of variation. (b) shows the interaction between blade shape and rotating speed affects the coefficient of variation. (c) shows the interaction between blade shape and height affects the coefficient of variation

The regression model reflects the influence of rotating speed, height, blade shape on variable coefficient (temperature stability), the higher the variable coefficient is, the greater temperature change would be, and that is not good for pasteurization of normal milk. Table 3 shows that the influence order of all factors on variable coefficient is as follows: shape > height > rotating speed. The response surface of influence of the interaction among factors on variable coefficient is shown in Figure 8a,b,c. The interaction effect between rotating speed and height was significant, while the interaction effects between height and shape, shape and rotating speed were not significant.

Figure 8a shows that, with the increase of height from 20 to 40 mm, the variable coefficient reduced before an increase; when the height reached 30 mm, the variable coefficient presented the minimum, at this time, the temperature had best stability; when rotating speed increased from 25 to 45 r/min, the variable coefficient reduced before an increase; when rotating speed reached 35 r/min, the variable coefficient reached the maximum value, and at this time, the temperature difference in the temperature field of the mixing system was the least, showing best temperature stability; Figure 8b,c shows the response surface of interaction effects between blade shape, rotating speed, and height, which were consistent in variation tendency. It can be seen that, among the three kinds of blades, the trapezoid frame had greatest influence on temperature stability, full trapezoid showed best effects, and with the increase of rotating speed and height, the variable coefficient presented a tendency of a decrease before an increase.

### 3.3 Optimization results of the RSM

To ensure the stability of temperature difference in the temperature field of the mixing system, namely small value of variable coefficient, the Optimization‐Numerical module in the software Design‐Expert 9 was applied for optimized solution on the regression equation model, and the constraint condition for optimization was as follows: objective function min *Y* (*X*_1_,* X*_2_, *X*_3_); variable range 25 ≤ *X*_1_ ≤ 45, 20 ≤ *X*_2_ ≤ 40, −1 ≤ *X*_3_ ≤ 1. The optimal parameter combination was obtained after optimization as follows: rotating speed was 30.28 r/min, height was 30.98 mm, the blade shape was −0.92, and the theoretical coefficient of variation after optimization was 0.0046, which is lower than 0.0054 of the No. 10 test in Table 2. The optimal parameter combination after rounding was as follows: rotating speed was 30 r/min, the height was 31 mm, and blade shape was 1 (full trapezoidal).

### 3.4 Optimization results of the BP‐GA model

**Figure 9:**
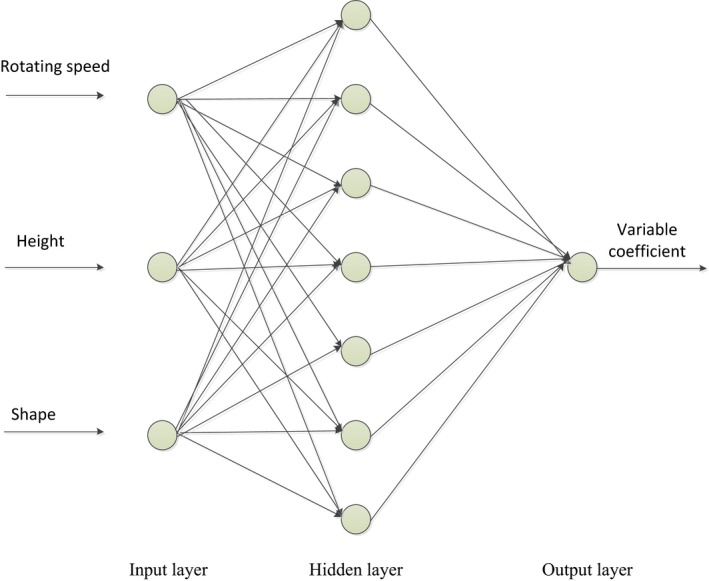
Topology diagram of neural network. Rotating speed, blade shape and height were input values, and variable coefficient was the output value

BP‐GA model optimization method was applied for optimization analysis on the performance stability during material processing. In the optimization process, firstly, two of 20 groups of data corresponding to 20 time points were randomly extracted under each factor level combination. There were a total of 17 factor level combinations in the test, that is to say, a total of 34 sets of data were extracted, and then, the 34 sets of data were taken as training set for learning the training by BP neural network. The 17 sets of test results in the table were taken as test set to evaluate the prediction performance of the neural network. The neural network structure had three layers, which are input layer neurons, hidden layer neurons and output layer neurons, the transfer function of hidden layer and output layer were tansig function and Purelin function, respectively, which determined that the number of neurons in input layer was 3, number of neurons in output layer was 1. The number of neurons in hidden layer was in the range of 3–12, as calculated based on Equation 9. According to the analysis and comparison of the results of previous program running, the best number of neurons in the hidden layer was finally determined to be 7. According to test arrangement, rotating speed, blade shape, and height were input values, and variable coefficient was the output value (the topological diagram is shown in Figure 9). As the program ran, the *X*_3_ blade shapes of full trapezoidal, full rectangular, and trapezoid frame were numbered 1, 2, and 3 for replacement.

(9) $$N=\begin{array}{cc} \sqrt{L_{\text{in}}+L_{\text{out}}}+a; & a\in 1-10 \end{array}$$

where *N* is the number of nodes in the hidden layer, *L*_in_ is the number of nodes of input layer, and *L*_out_ is the number of nodes in the output layer.

**Figure 10:**
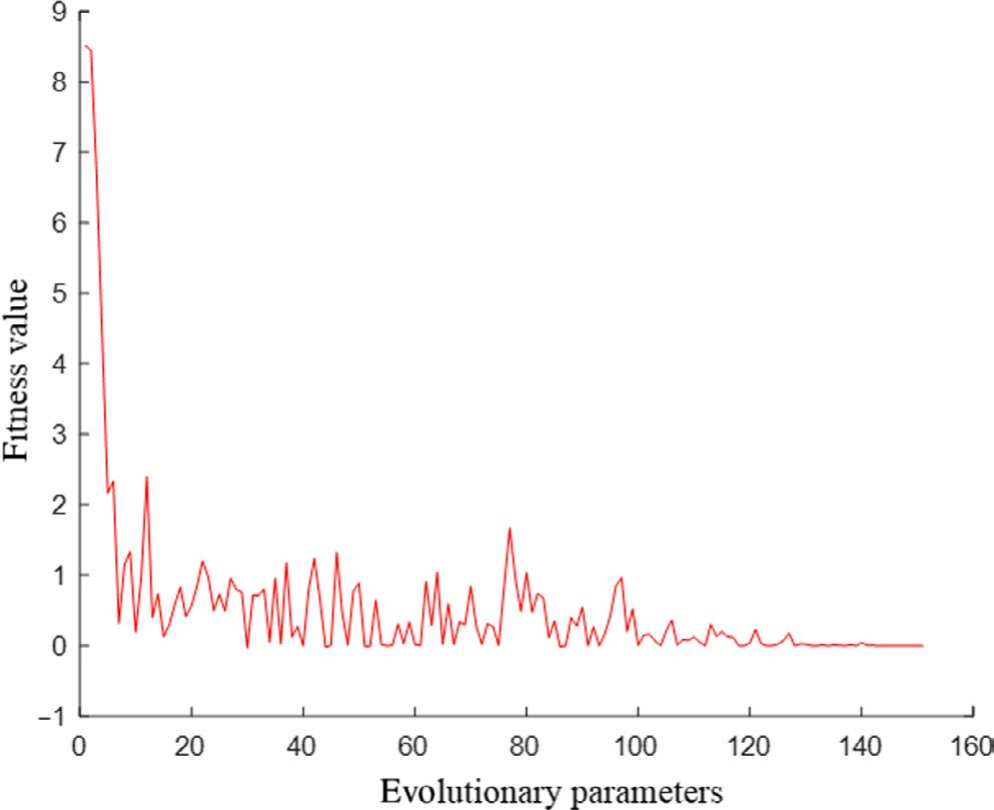
Variation curves of fitness value

The predicted output of trained BP neural network was taken as the individual fitness value for genetic algorithm optimization calculation, and the optimal values and corresponding input values of the function were found through selection, interlace operation, and mutation operations. The number of evolutionary parameters of genetic algorithm optimization is 150, the population size is 20, the crossover probability is 0.4, the mutation probability is 0.2, the floating point number is encoded, the individual length is 3, the individual parameters mainly include the individual fitness function trained by BP neural network, the population size evolution algebra, the variable function, the optimal fitness value, and the optimal individual of each generation of the population. In the process of BP‐GA algorithm, the curve of fitness value with change of evolutionary parameters is shown in Figure 10. It can be seen that after 130 iterations, the fitness value has basically reached stability. The optimal fitness value obtained by BP‐GA grid optimization is 0.0036. The corresponding function position is (33.8175, 25.3945, 1.0202), that is, when the rounded variable factor level value is the speed of 34 r/min, the height of 25 mm, and the shape is 1 (trapezoidal full face), the minimum coefficient of variation of temperature change stability is 0.0036.

### 3.5 Comparison of RSM and BP‐GA optimization methods

**Figure 11:**
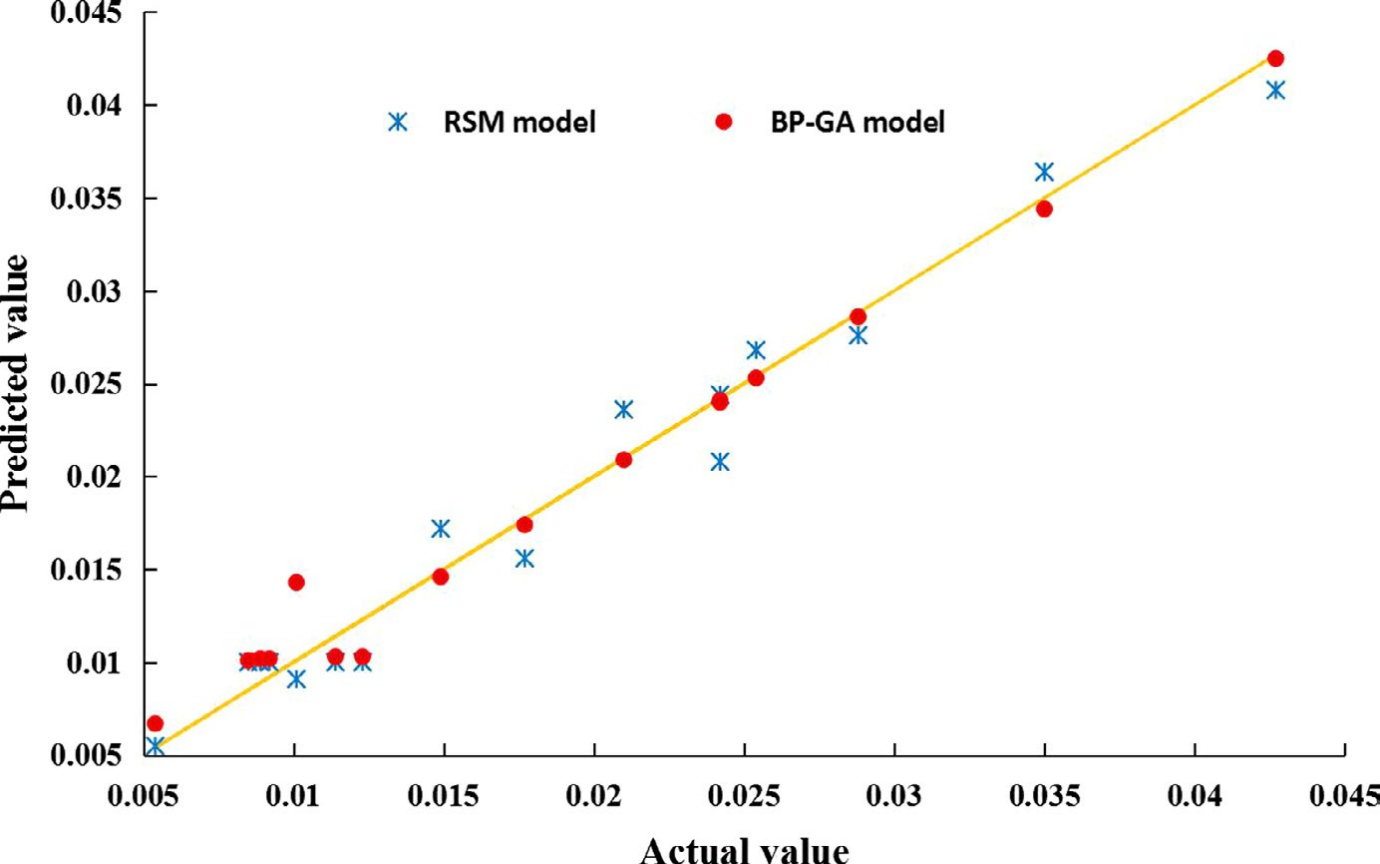
Predicted scatter diagram of RSM and BP‐GA models. The prediction ability of BP‐GA neural network model was better than the RSM model

The prediction ability of models can be evaluated by predicting root‐mean‐square error RMSEP, determination coefficient *R* ^2^, and relative percent difference (RPD; Dai, Cheng, Sun, Zhu, & Pu, 2016; Dong, Zhao, et al., 2017; Funes, Allouche, Beltran, Aguliera, & Jimenez, 2018; Wu et al., 2019). The lower the predicted root‐mean‐square error is, the higher the determination coefficient and relative percent difference would be, and higher prediction precision and generalization of the model would be (Guo et al., 2010; Magwaza, Naidoo, Laurie, & Shimelis, 2016). The regression model prediction was carried out according to the regression Equation 8, and the running prediction of the BP‐GA optimization algorithm program was carried out, and the results are shown in Table 2. Table 2 and Figure 11 show that the root‐mean‐square error predicted by BP‐GA neural network prediction model was 0.0013, determination coefficient was 0.9960, and relative percent difference was 8.4961, which were better than the evaluation indexes of RSM model, showing that the prediction ability of BP‐GA neural network model was better than the RSM model. In addition, Table 4 shows that the variable coefficient value predicted by BP‐GA neural network was 0.0036, test value was 0.0035, with relative error of 2.9%. The optimal value of the response surface method after quadratic polynomial fitting was 0.0046, the test value was 0.0044, and the relative error was 4.5%, indicating that the extreme value predicted by the response surface method was not really the minimum coefficient of variation, and the optimized value predicted by BP‐GA neural network algorithm was more closer to the extreme value.

**Table 4:** Comparison of RSM and BP‐GA optimization methods

Methods	*X*_1_/r/min	*X*_2_/mm	*X*_3_	*Y*	Predicted values of RSM	Predicted values of BP‐GA	Relative error, %
RSM	30	31	Full trapezoid	0.0044	0.0046		4.5
BP‐GA	34	25	Full trapezoid	0.0035		0.0036	2.9

## 4 CONCLUSIONS

In this paper, temperature stability was taken as the evaluation index for processing performance. Taking the rotating speed of agitator blades, the height from blades to the barrel bottom and shape of the agitator blades as variables, a test design was conducted to study the influence order of the factors on temperature stability, which was shape > height > rotating speed. The comparison between the optimization results of RSM and BP‐GA neural network algorithms showed that, in the optimization process by RSM, when rotating speed was 30 r/min, height was 31 mm, blade shape was a full trapezoid, and the predicted value and actual value of variable coefficient were 0.0046 and 0.0044, respectively, with relative error of 4.5%. In the optimization by BP‐GA neural network algorithm, when rotating speed was 34 r/min, height was 25 mm, blade shape was a full trapezoid, and the predicted value and actual value of variable coefficient were 0.0036 and 0.0035, respectively, with relative error of 2.9%.

It is feasible to optimize parameter combination by applying RSM and BP‐GA neural network algorithm. The predicted root‐mean‐square error of the model by the BP‐GA neural network algorithm was 0.0013, determination coefficient was 0.9960, and relative percent deviation was 8.4961, which showed better performance than the RSM model. Therefore, in the parameter optimization of the mixing device for constant milk processing, the BP‐GA neural network algorithm has better fitting performance than the RSM, and then, the optimal working parameter combination in the range of global variable was as follows: rotating speed was 34 r/min, height was 25 mm, and blade shape was a full trapezoid, which could provide reference to improving normal milk processing and mixing device design and normal milk processing quality.

## CONFLICT OF INTEREST

The authors declare that we do not have any conflict of interest.

## ETHICAL APPROVAL

This study does not involve any human or animal testing. Written informed consent was obtained from all study participants.
